# Electrocardiogram characteristics of P wave associated with successful pulmonary vein isolation in patients with paroxysmal atrial fibrillation: Significance of changes in P‐wave duration and notched P wave

**DOI:** 10.1111/anec.12712

**Published:** 2019-09-30

**Authors:** Satoshi Yanagisawa, Yasuya Inden, Hiroya Okamoto, Aya Fujii, Yusuke Sakamoto, Keita Mamiya, Toshiro Tomomatsu, Rei Shibata, Toyoaki Murohara

**Affiliations:** ^1^ Department of Advanced Cardiovascular Therapeutics Nagoya University Graduate School of Medicine Nagoya Japan; ^2^ Department of Cardiology Nagoya University Graduate School of Medicine Nagoya Japan

**Keywords:** atrial fibrillation, catheter ablation, notched P wave, P‐wave duration, signal‐averaged electrocardiogram

## Abstract

**Background:**

The mechanisms involved in changes in P wave following catheter ablation for atrial fibrillation (AF) are uncertain. This study aimed to assess the relationship between changes in P‐wave morphology and pulmonary vein (PV) reconnection following ablation by the assessment of 12‐lead surface electrocardiogram and signal‐averaged electrocardiogram.

**Methods:**

This retrospective study included 115 consecutive patients with paroxysmal AF that underwent repeat ablation for recurrence following initial ablation. We investigated changes in P‐wave morphology between baseline and repeat procedure in patients with and without PV reconnection. The study also included as validation group without recurrence (*n* = 67) following initial ablation.

**Results:**

The maximum P‐wave duration (PWD) was significantly decreased from baseline to just after the procedure in all groups. However, for the PV reconnection group (*n* = 100), the maximum PWD was significantly increased again at the repeat procedure. In contrast, the maximum PWD was significantly reduced between baseline and repeat procedure in the non‐PV reconnection group (*n* = 15). The signal‐averaged PWD was significantly decreased from baseline to repeat procedure in the non‐PV reconnection group, but, conversely, was increased in the PV reconnection group. In the non‐PV reconnection group, the disappearance of notched P wave was detected in 8 of 15 patients (53%), which was significantly higher than in other groups (*p* = .001). A new or delayed notched P wave was identified in the PV reconnection group only. These results were confirmed in the validation group.

**Conclusions:**

The reverse dynamics of PWD after initial shortening directly following ablation were significantly associated with PV reconnection.

## 1 INTRODUCTION

Catheter ablation therapy is an established treatment for patients with paroxysmal atrial fibrillation (AF), and its success rate is higher than 70%–80% after the single procedure (Calkins et al., 2017). The primary goal of catheter ablation for paroxysmal AF is the achievement of complete electrical isolation of all pulmonary veins (PVs), because electrically isolated ectopic activity in PV plays an important role in initiating and perpetuating AF (Haissaguerre et al., 1998). Recurrence after ablation is considered the reconnection of electrical conduction between the PV and left atrium (LA) in patients with paroxysmal AF. During a repeat procedure for recurrence cases, there may be multiple PV reconnections, despite the development of advanced ablation techniques and devices (Kim et al., 2017; Shah et al., 2018; Zucchelli et al., 2018). Even reconnection of one PV with a critical arrhythmogenic substrate can be responsible for the recurrence of AF following ablation. However, the presence or absence of PV reconnection could be confirmed only by a repeat procedure in which an electrode catheter is inserted into the LA and PV invasively. There is no standard noninvasive examination to confirm durable PV isolation after the ablation.

The P wave on the electrocardiogram (ECG) represents atrial activation. The P‐wave morphology largely depends on the LA activation vector, which may be defined by the localization of the LA breakthrough site, the sinus rhythm origin defining the right atrial depolarization vector, and the size of the atrial chambers (Platonov, 2012). Several studies reported a possible involvement of LA‐PV junction and terminal PV sleeve potential in the P‐wave morphology by simulation or virtual methods (Ogawa et al., 2007; Okumura et al., 2007). Previous studies have also reported that P‐wave duration (PWD) and morphology were significantly changed after successful PV isolation for AF, while some investigators speculated that the elimination of the myocardial sleeve of PVs may contribute changes in PWD and morphology (Blanche et al., 2014; Blanche, Tran, Rigamonti, Burri, & Zimmermann, 2013; Caldwell et al., 2014; Kanzaki et al., 2016; Nakatani et al., 2016; Ogawa et al., 2007; Okumura et al., 2007; Van Beeumen, Houben, Tavernier, Ketels, & Duytschaever, 2010). However, the majority of these studies focused on association between changes in the P wave and clinical outcome, recurrence or nonrecurrence. A detailed assessment of P‐wave morphology with PV reconnections is lacking. We hypothesized that the reduction in the PWD is characterized by the presence of residual conduction between the LA and PV after the ablation, and that there are specific and significant changes in P‐wave morphology in patients with or without PV reconnection after the ablation. Thus, the present study focused on patients with paroxysmal AF who underwent repeat procedures for recurrence after an initial ablation. We assessed the relationship between the PV reconnection and changes in the P wave by evaluating 12‐lead surface ECG and signal‐averaged electrocardiogram (SAECG) results, in comparison with those obtained at the first preablation in same patient.

## 2 METHODS

### 2.1 Study population

**Figure 1:**
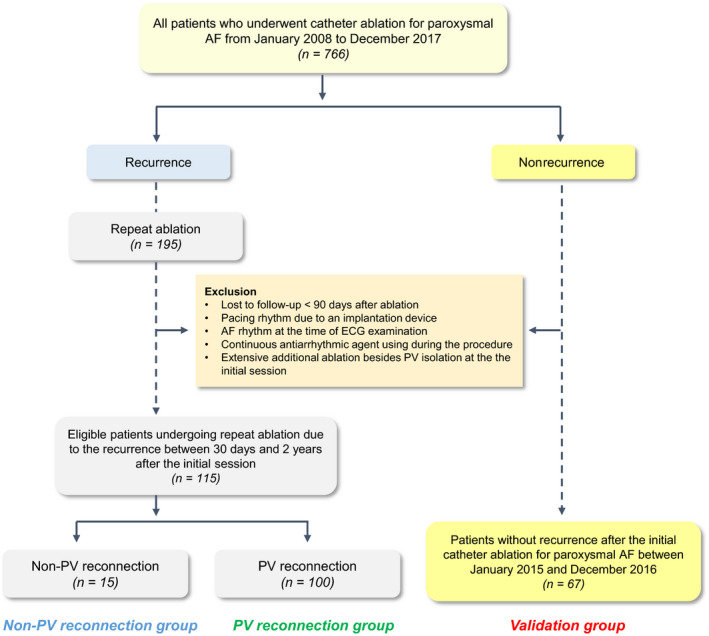
Flowchart of the study design. AF, atrial fibrillation; ECG, electrocardiogram; PV, pulmonary vein

The study population was retrospectively analyzed from a catheter ablation database at Nagoya University Hospital, Japan. This study was approved by our institutional ethics committee. Among the 766 consecutive patients who underwent catheter ablation for paroxysmal AF between January 2008 and December 2017 at Nagoya University Hospital, 195 patients were subjected to a repeat ablation for recurrence after the initial session. A total of 115 consecutive patients undergoing PV isolation only in the LA at the first session and repeat ablation between 30 days and 2 years after the initial session were included in the study population (Figure 1). The population was divided into a non‐PV reconnection group and a PV reconnection group based on the findings at the repeat procedure. All patients were completely examined with a 12‐lead ECG under during sinus rhythm the day before and just after the first session, and the day before the repeat session; all patients were examined using SAECG during sinus rhythm the day before the first session and at the repeat session. Patients with pacing rhythm due to an implantation device, AF rhythm at the time of ECG examination, and continuous antiarrhythmic agent use during the procedure were excluded. In addition, we selected a validation cohort group with consecutive patients (*n* = 67) who underwent catheter ablation for paroxysmal AF between January 2015 and December 2016 in our institution, and who were free from recurrence after the initial ablation (Figure 1). The validation group was fully examined using the 12‐lead ECG under sinus rhythm the day before, just after the procedure, and 6 months after the first ablation. SAECG was performed the day before and 1 month after the ablation. The indications for catheter ablation for AF were in compliance with the most recent guideline (Calkins et al., 2017). Antiarrhythmic agents were discontinued at least five half‐lives of these drugs before the ablation. Prior to the procedure, informed consent was obtained from all patients based on our hospital guideline. This study was performed in compliance with the principles of the Declaration of Helsinki.

### 2.2 Catheter ablation procedures

Patients who were scheduled for catheter ablation treatment were admitted the day before the procedure. At admission, baseline blood testing, echocardiography, and electrocardiography were performed.

Three ablation techniques were applied to the study population: radiofrequency ablation in 87, cryoballoon ablation in 27, and Hot balloon ablation in 1 patient(s). The attending physician determined which ablation application should be performed for each patient. As for the radiofrequency ablation procedure, encircling PV isolation was performed with a 3.5‐mm tip, open‐irrigated ablation catheter (NaviStar ThermoCool, Biosense Webster, Inc.) using a circular mapping catheter placed on the ostium of PV atrium. All ablation procedures were performed using a three‐dimensional electroanatomical mapping system. The radiofrequency energy output was titrated to 25–35 W at a flow rate of 17–30 ml/min, with a maximum temperature of 42°C. The irrigated contact force (CF) sensing catheter (NaviStar ThermoCool SmartTouch, Biosense Webster, Inc.) was available in Japan since 2013. Thereafter, most radiofrequency ablation cases underwent the ablation procedure using the irrigated CF sensing catheter. During the ablation, catheter stability was set with a target force‐time integral of >100 g*s (Yanagisawa et al., 2018). The CF was targeted as 10–20 g, and a maximum CF threshold of 50 g was set for all cases. The radiofrequency energy was continuously applied from the start of the ablation point to the end of the encircling PV. However, the duration of ablation in each point was maintained at approximately 20–30 s.

For the cryoballoon ablation procedure, a second‐generation 28‐mm cryoballoon system (Arctic Front Advance, Medtronic) was advanced and placed on the ostium of each PV using an inner circular mapping catheter (Achieve, Medtronic). After confirming the PV ostium occlusion with the cryoballoon, a 120–180 s cycle freeze ablation was repeated until the electrical isolation of PV was achieved.

The Hot balloon ablation procedure has been introduced and available since April 2016 in Japan (Sohara et al., 2016). The balloon was placed into the target PV ostium while advancing the guidewire through a catheter lumen into the PV. Thereafter, all PVs were ablated consecutively. The ablation time was set as 150–240 s for one cycle, which was repeated to confirm PV isolation. Touch‐up radiofrequency ablation was used for a residual potential of the PV after several attempts of Hot balloon ablation.

We included only patients undergoing PV isolation in LA ablation in the analysis. If the rhythm of the patients did not convert to sinus rhythm at the end of ablation, external cardioversion was performed. The endpoint of the ablation procedure was a confirmation of bi‐directional PV isolation. In the initial session, intravenous isoproterenol infusion and adenosine test were not routinely used after confirming PV isolation.

In the repeat procedure, all ablations were performed by the application of radiofrequency ablation. We first checked the PV reconnection for all PVs using circular mapping catheter. If a PV reconnection was found, a subsequent ablation was applied to isolate the PV. After the confirmation of complete PV isolation, we evaluated non‐PV ectopic beats and AF occurrence with intravenous isoproterenol infusion or rapid atrial stimulation.

### 2.3 Twelve‐lead surface ECG analysis

A standard 12‐lead surface ECG was obtained the day before the first and repeat ablation procedures. The ECG was digitally recorded with a paper speed and a scale set at 25 mm/s and 10 mm/mV, respectively (Cardio Star, FCP‐7541; Fukuda Denshi). All intervals and parameters of the 12‐lead ECG were measured using a digital caliper in high amplification (more than 20 mm/mV and 50 mm/s). The PWD was measured as the maximum PWD of the 12‐lead surface ECG recording. The onset of the P wave was determined as the initial upward or downward deflection from the isoelectric line. The off‐set of the P wave was identified as the returning point of the isoelectric line. The isoelectric line was determined from the beginning of the PQRS complex to the end of the T‐wave.

In addition, we measured the PWD on the 12‐lead surface ECG just after the procedure using the EP WorkMate Recording system (St. Jude Medical). Electrocardiographic recordings were amplified to 0.3 mV/cm with a sweep speed of 150 mm/s. Measurement of the PWD was performed in the same manner as described above.

These parameters were calculated by the investigator who was blinded to the outcomes of PV reconnection. Additionally, we randomly obtained 276‐lead ECGs with 23 patients among the study population. The ECG parameters were measured by two investigators who were blinded to the outcomes to test a reliability and reproducibility for measurement. The intra‐ and interinvestigator correlations of the maximum PWD were 0.656 and 0.673 (*p* < .001), respectively. The Bland–Altman analyses indicated that the mean difference with 95% limits of agreement in the maximum PWD between the intra‐ and interinvestigators was −1.87 (−5.09 to 1.35) ms (*p* = .241) and −3.66 (−7.53 to 0.20) ms (*p* = .062), and the regression line slope was *r* = −.160 (*p* = .466) and *r* = −.301 (*p* = .163), respectively.

The P‐wave morphology (appearance and/or disappearance of notches) analyses were manually performed using the 12‐lead surface ECG amplified with the 20 mm/mV and 50 mm/s setting. The two investigators checked and agreed upon the presence and diminishment of the notch in the P wave.

### 2.4 Signal‐averaged P‐wave electrocardiogram analysis

A signal‐averaged P‐wave electrocardiogram was recorded the day before the first and repeat ablation procedures concurrently with the 12‐lead surface ECG recording. The basic methodology of SAECG used at our institution has been was described previously (Kanzaki et al., 2016). The P wave of the SAECG was recorded in the P‐wave‐triggered mode (Cardio Star; Fukuda Denshi Co.). By applying a P‐wave recognition program to eliminate extra systole, a signal of >250 beats was averaged from a standard 12‐lead ECG and the noise amplitude was reduced to <0.5 μV. The signal from each lead was filtered bidirectionally (with forward and backward filters) through a filter setting between 40 and 300 Hz. The corrected filtered PWD was used for the analysis in this study. Finally, automatic measurements were corrected according to the visual delineation of the beginning and the end of the P wave.

### 2.5 Follow‐up

Patients remained hospitalized under continuous rhythm monitoring for 3 days after the procedure. After discharge, patients were followed up through the outpatient clinic at minimum every month after ablation. At each follow‐up visit, patients underwent 12‐lead ECG and were asked about any symptoms related to the presence of arrhythmia. Twenty‐four hour Holter monitoring testing was also performed repeatedly, if necessary. Recurrence was defined as any AF or atrial tachycardia of more than 30 s in duration. All patients undergoing repeat ablation procedures were identified of any AF recurrence by the abovementioned examinations after the initial session.

### 2.6 Statistical analysis

Differences in the baseline characteristics between different subgroups were analyzed using a one‐way analysis of variance for continuous variables and the chi‐squared test for dichotomous variables, as appropriate. Differences between the baseline and follow‐up in outcome parameters were compared using a paired *t* test. To test for a significant difference in means over time on the same subjects, a repeated measures analysis of variance was used. Bonferroni correction was applied for post hoc multiple comparisons. Based on the obtained parameters, a receiver operating characteristic (ROC) curve was generated, and the cutoff point for ROC curve factor was determined. The intraobserver and interobserver reliability for the measurement of the ECG parameters was examined using intraobserver and intercorrelation coefficients. Correlations between two samples were made using Spearman's rank correlation coefficient. The Bland–Altman difference plots with 95% confidence limits were constructed to evaluate the degree of agreement between the two measurements. The 95% limits of agreement were calculated as the mean difference ± 2 standard deviation. A *p*‐value of <.05 was considered statistically significant.

## 3 RESULTS

### 3.1 Baseline patient characteristics

Among the patients who underwent repeat ablation for recurrence, 15 and 100 were classified into the non‐PV reconnection and PV reconnection groups, respectively. The duration between the initial session and repeat procedure was 207 ± 159 days. At the repeat ablation procedure, the mean number of PV reconnections identified was 2.1 ± 1.2 PVs. PV reconnections occurred as follows: 1 PV in 17 patients, 2 PVs in 40, 3 PVs in 28, and 4 PVs in 15. The mean duration between recurrence of AF and the repeat session was 73 ± 89 days. The baseline characteristics and examination results among the non‐PV reconnection, PV reconnection, and validation cohort groups are shown in Table 1. There were significant differences in duration of AF, left ventricular ejection fraction, and prevalence of radiofrequency ablation among the groups.

**Table 1:** Comparison of demographic and baseline characteristics among the non‐PV reconnection, PV reconnection, and validation groups

Parameters	Non‐PV reconnection (*n* = 15)	PV reconnection (*n* = 100)	Validation group (*n* = 67)	*p*‐value
Age, year	65.3 ± 11.3	63.0 ± 11.2	64.1 ± 9.9	.655
Male sex	8 (53%)	66 (66%)	46 (69%)	.527
Body mass index, kg/m^2^	24.2 ± 3.1	23.7 ± 3.3	24.9 ± 3.4	.080
Duration of AF, year	4.8 ± 5.7	3.0 ± 3.8	1.8 ± 3.3	.016
Symptoms	14 (93%)	88 (88%)	51 (76%)	.072
Number of antiarrhythmic drugs	1.0 ± 0.7	0.9 ± 0.9	0.7 ± 0.7	.276
Comorbidities				
Hypertension	6 (40%)	57 (57%)	33 (49%)	.534
Diabetes mellitus	3 (20%)	12 (12%)	10 (15%)	.660
Heart failure	1 (7%)	3 (3%)	1 (1%)	.527
Stroke	2 (13%)	11 (11%)	4 (6%)	.471
Echocardiographic data				
Left atrial diameter, mm	38.3 ± 6.3	37.4 ± 5.9	37.0 ± 5.1	.716
Left ventricular ejection fraction, %	66.3 ± 4.5	63.3 ± 5.6	65.6 ± 6.0	.017
CHADS2 score	1.2 ± 1.0	1.1 ± 1.0	1.0 ± 1.0	.549
CHA2DS2‐VASc score	2.4 ± 1.6	2.0 ± 1.5	2.0 ± 1.5	.573
Laboratory data				
CrCl level, mL/min	87.3 ± 39.0	85.3 ± 32.7	88.2 ± 28.2	.837
B‐type natriuretic peptide levels, pg/dL	98.0 ± 140.4	46.9 ± 60.0	36.5 ± 73.4	.017
First session of radiofrequency ablation for PV isolation	7 (47%)	80 (80%)	8 (11%)	<.001
Duration between initial and repeat session, days	243 ± 182	197 ± 170		.920
Maximum PWD before first ablation, mm	135.3 ± 13.2	128.6 ± 12.2	126.0 ± 16.2	.059
Signal‐averaged PWD before first ablation, mm	139.4 ± 16.2	130.9 ± 14.6	129.8 ± 14.7	.074

Note

The data are presented as numbers (%) and means ± standard deviations.

Abbreviations: AF, atrial fibrillation; CrCl, creatinine clearance; PV, pulmonary vein; PWD, P‐wave duration.

### 3.2 Changes in ECG parameters from the initial session to the repeat session

In patients undergoing repeat ablation for recurrence including the non‐PV reconnection and PV reconnection groups, a significant change was found in the maximum PWD (129.5 ± 12.8 vs. 125.9 ± 14.1 ms, *p* = .001) between the initial and repeat ablation sessions. However, no significant difference in PWD in SAECG (132.0 ± 15.0 vs. 133.5 ± 15.7 ms, *p* = .237) was observed at the repeat session, compared to that at the baseline.

**Figure 2:**
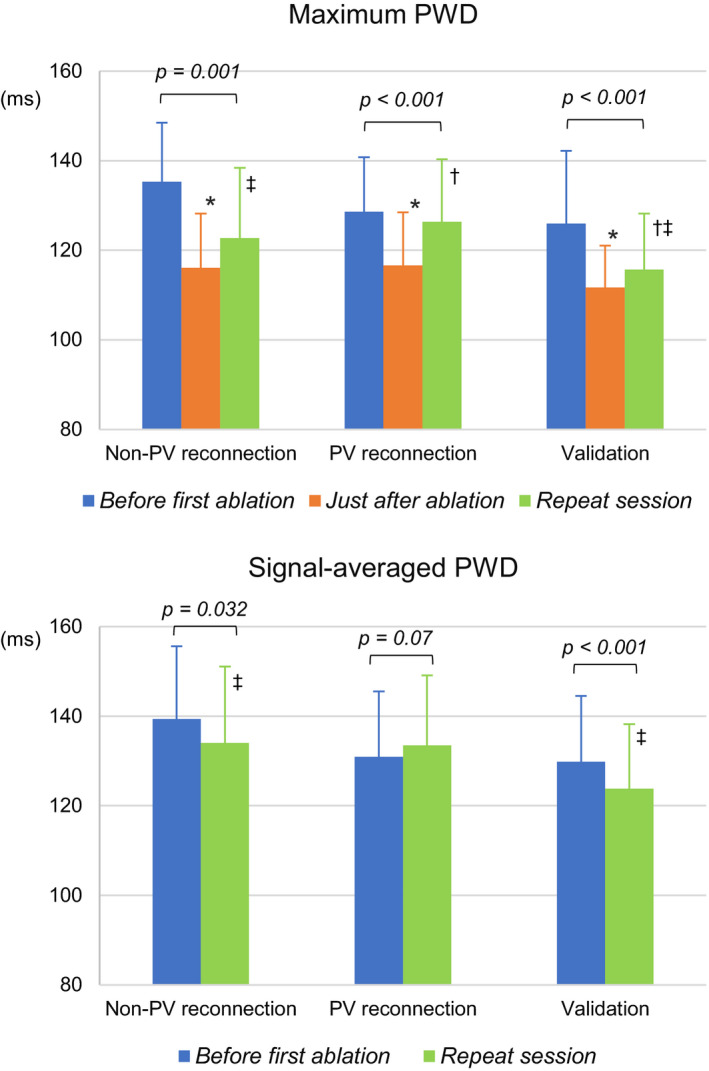
Changes in electrocardiogram parameters between the initial and repeat procedures. *p < .05 baseline versus just after the procedure. †*p* < .05 just after the procedure versus repeat procedure. ‡*p* < .05 baseline versus repeat procedure. PV, pulmonary vein; PWD, P‐wave duration. Note that the follow‐up ECG and signal‐averaged ECG analyses in the validation cohort group were obtained 6 and 1 month after the first ablation, respectively

Changes in ECG parameters between the baseline and repeat procedure among the non‐PV reconnection, PV reconnection, and validation cohort groups are shown in Table 2 and Figure 2. The maximum PWD was significantly decreased from baseline to just after the procedure in all groups. However, for the PV reconnection group, the maximum PWD was significantly increased again at the repeat procedure. When comparing the baseline and repeat procedure, the maximum PWD was significantly reduced in the non‐PV reconnection group (135.3 ± 13.2 vs. 122.7 ± 15.7 ms, *p* = .001); however, this change was not observed in the PV reconnection groups. In the validation cohort group, significant decreases in the maximum PWD from baseline to just after the ablation and in the maximum PWD from baseline to 6 months after the ablation were found. The PWDs in the SAECG were significantly decreased between baseline and the repeat procedure in the non‐PV reconnection group (139.4 ± 16.2 vs. 134.0 ± 17.1 ms, *p* = .032), and between baseline and 1 month after the ablation in the validation cohort group (129.8 ± 14.7 vs. 123.8 ± 14.4 ms, *p* < .001). In contrast, in the PV reconnection group, the PWD in SAECG increased at the repeat procedure, compared to that at the baseline (130.9 ± 14.6 vs. 133.5 ± 15.6 ms, *p* = .07).

**Table 2:** Changes in electrocardiogram parameters between the baseline and repeat procedures

	Baseline	Just after the procedure	Repeat procedure	*p*‐value
P‐wave duration (maximum)				
Non‐PV reconnection group	135.3 ± 13.2 ms	116.1 ± 12.1 ms*	122.7 ± 15.7 ms‡	.001
PV reconnection group	128.6 ± 12.2 ms	116.6 ± 11.9 ms*	126.4 ± 13.9 ms†	<.001
Validation group	126.0 ± 16.2 ms	111.7 ± 9.3 ms*	(115.7 ± 12.5 ms†‡)	<.001
P‐wave duration (signal‐averaged electrocardiogram)				
Non‐PV reconnection group	139.4 ± 16.2 ms	n/a	134.0 ± 17.1 ms‡	.032
PV reconnection group	130.9 ± 14.6 ms	n/a	133.5 ± 15.6 ms	.070
Validation group	129.8 ± 14.7 ms	n/a	(123.8 ± 14.4 ms‡)	<.001

Note

Note that the follow‐up ECG and signal‐averaged ECG analyses in the validation cohort group were obtained 6 and 1 month after the first ablation, respectively.

Abbreviation: PV, pulmonary vein.

* *p* < .05 baseline versus just after the procedure.

^†^ *p* < .05 just after the procedure versus repeat procedure.

^‡^ *p* < .05 baseline versus repeat procedure.

The reconnection of the left superior, left inferior, right superior, and right inferior PVs was 60, 43, 74, and 64 PVs, respectively. Changes in electrocardiogram parameters in the left and right PV reconnection groups are shown in Table 3. The left PV reconnection group showed a trend toward a greater increase in signal‐averaged PWD at the repeat procedure (*p* = .062).

**Table 3:** Changes in electrocardiogram parameters in the left and right PV reconnection groups

	Baseline	Just after the procedure	Repeat procedure	*p*‐value
P‐wave duration (maximum)				
Left PV reconnection (*n* = 70)	130.1 ± 12.5 ms	116.8 ± 12.5 ms*	128.4 ± 14.5 ms†	<.001
Right PV reconnection (*n* = 84)	127.9 ± 12.4 ms	117.1 ± 11.7 ms*	126.0 ± 14.0 ms†	<.001
Both left and right PVs reconnection (*n* = 54)	129.3 ± 13.0 ms	117.6 ± 12.5 ms*	128.3 ± 14.9 ms†	<.001
P‐wave duration (signal‐averaged electrocardiogram)				
Left PV reconnection (*n* = 70)	132.2 ± 14.5 ms	n.a.	135.4 ± 15.2 ms	.062
Right PV reconnection (*n* = 84)	131.0 ± 14.5 ms	n.a.	133.0 ± 15.2 ms	.189
Both left and right PVs reconnection (*n* = 54)	132.7 ± 14.3 ms	n.a.	135.2 ± 14.6 ms	.193

Abbreviation: PV, pulmonary vein.

* *p* < .05 baseline versus just after the procedure.

^†^ *p* < .05 just after the procedure versus repeat procedure.

### 3.3 Focus on the ECG changes in the non‐PV reconnection group

**Figure 3:**
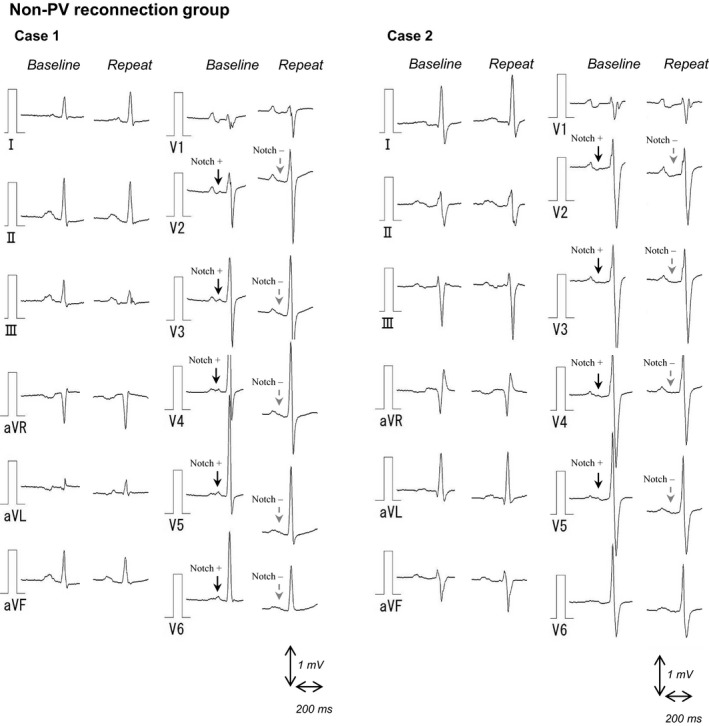
Changes in P‐wave morphology on the electrocardiogram following ablation in patients without PV reconnection at the time of repeat procedure. In case 1, second peak of the P wave after a notch presented at the V2 through V6 lead on initial ablation. At the repeat procedure, these second portions of the P wave including the notch were absent. Similarly, in case 2, a discrete second part of P wave after a notch was identified in the V2‐V6 leads at baseline, and then, these later portions of the P wave and notch disappeared at the time of repeat session. PV, pulmonary vein

By means of careful observation of the individual 12 leads of the surface ECG at baseline and repeat ablation, we identified a specific change in P‐wave morphology after ablation, particularly in the precordial leads. In case 1 presented in Figure 3, the second peak of the P wave after a notch was presented at the V2 through the V6 lead at the initial ablation. At the repeat procedure, these second portion of the P wave including the notch was eliminated. Similarly, in case 2 in Figure 3, a second peak of the P wave after a notch was identified in the V2–V6 leads at baseline, and at the repeat session, these later parts of the P wave and notch disappeared. Additional cases (case 3–8) in the non‐PV reconnection group are presented in Figure S1. All cases showed similar changes in P‐wave morphology (disappearance of the notched P wave) in the precordial leads, except for case 8 in the inferior leads. We noticed this specific change in morphology in 8 of 15 patients (53%) in the non‐PV reconnection group, which was significantly higher than that observed to the PV reconnection group and validation cohort group (12 patients [12%] and 14 patients [21%]; *p* = .001) (Table 4).

**Table 4:** The appearance or disappearance of notched P wave on the electrocardiogram among the non‐PV reconnection, PV reconnection, and validation groups

	Non‐PV reconnection (*n* = 15)	PV reconnection (*n* = 100)	Validation group (*n* = 67)	*p*‐value
Disappearance of notched P wave	8 (53%)	12 (12%)	14 (21%)	.001
A new or delayed notched P wave	0 (0%)	16 (16%)	5 (8%)	.082

Abbreviation: PV, pulmonary vein.

### 3.4 Focus on the ECG changes in the PV reconnection group

**Figure 4:**
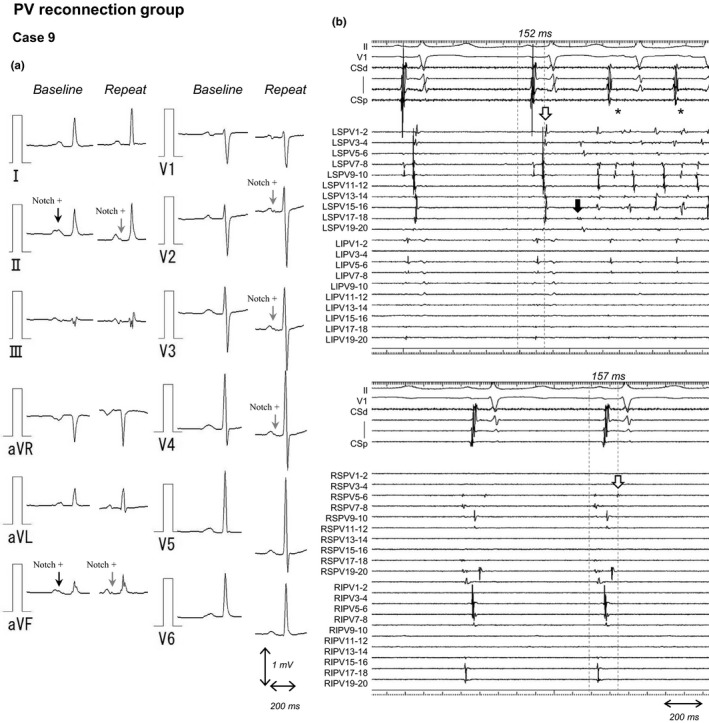
Changes in P‐wave morphology on the electrocardiogram following ablation and intracardiac electrogram at the repeat procedure in a patient presenting all 4 PVs reconnection. (a) In case 9, the 12‐lead surface electrocardiogram at the initial ablation demonstrated the presence of a second portion of P wave after a notch in the inferior leads. In the repeat procedure, the electrocardiogram showed a new notched P wave in the V2‐4 leads. In addition, the second part of the P wave at the inferior leads at the baseline showed a delay. (b) On the intracardiac electrogram at the time of the repeat procedure in the same patient, the potential of the LS and RSPVs recorded in the circular mapping catheter positioned at the PV‐LA junction resulted to be on a significant delay (152 and 157 ms, respectively) from the beginning of P wave (white arrow). Notably, the AF occurred from the LSPV (black arrow), but the LA including the LIPV presented a slow regular atrial tachycardia indicating a conduction delay between the LSPV and LA (*). CS, coronary sinus; LA, left atrium; LIPV, left inferior pulmonary vein; LSPV, left superior pulmonary vein; PV, pulmonary vein; RIPV, right inferior pulmonary vein; RSPV, right superior pulmonary vein

Figure 4 represents a case in which all 4 PVs reconnection occurred at the repeat procedure. Twelve‐lead surface ECG at the initial ablation demonstrated the presence of a second part of the P wave after a notch in the inferior leads. In the repeat procedure, the ECG showed a new appearance of the discrete P wave and notch in the V2‐4 leads. In addition, the second part of the P wave in the inferior leads at baseline was markedly delayed. On the intracardiac electrogram at the time of the repeat procedure in this case, the potential of the left superior and right superior PVs recorded by a circular mapping catheter positioned at the PV‐LA junction showed a significant delay from the beginning of P wave on the surface lead ECG (Figure 4b). We observed this specific morphological change of the new or delayed notched P wave in 16 patients (16%) in the PV reconnection group. However, we did not find this phenomenon in the non‐PV reconnection group (Table 4).

### 3.5 ROC analyses of PWD for prediction of non‐PV reconnection

The cutoff value for the shortening of the maximum PWD for non‐PV reconnection based on a ROC curve analysis was 5.0 ms (area under the curve 0.765, 95% confidence interval 0.66–0.88) with a specificity of 87% and sensitivity of 59% (Figure S2), whereas the cutoff value for the improvement of the PWD in SAECG for non‐PV reconnection was 3.5 ms (area under the curve 0.688, 95% confidence interval 0.56–0.81) with a specificity of 73% and sensitivity of 66%. The prevalence in the decreased maximum PWD ≥ 5.0 ms and PWD in SAECG ≥ 3.5 ms after the ablation in the validation group was 72% and 66%, respectively. Combined parameters including a decrease in PWD and signal‐averaged PWD and changes in the P‐wave morphology (disappearance of the notched P wave) could enhance the positive predictive value for confirming the non‐PV reconnection after the ablation (Table 5).

**Table 5:** Predictive values for confirming a non‐PV reconnection after the ablation

	Sensitivity (%)	Specificity (%)	Positive predictive value (%)
(1) Decreased maximum PWD ≥ 5.0 ms	59	87	24
(2) Decreased SAPWD ≥ 3.5 ms	66	73	24
(1) + (2)	80	67	33
(3) Absence or disappearance of a notched P wave	88	53	40
(1) + (2) + (3)	97	33	63

Abbreviations: PV, pulmonary vein; PWD, p‐wave duration; SAPWD, signal‐averaged p‐wave duration.

### 3.6 Changes in echocardiography from the initial session to the repeat session

There were no significant changes in the LA diameter on the echocardiography between the initial and repeat procedures in the non‐PV reconnection group (from 38.3 ± 6.3 to 36.7 ± 5.9 mm, *p* = .167) and the PV reconnection group (from 37.4 ± 5.9 to 37.5 ± 5.6 mm, *p* = .826).

## 4 DISCUSSION

The PWD on ECG represents total atrial depolarization and reflects an intra‐ and interatrial conduction time. A prolonged PWD is considered to be a marker of electrical and structural remodeling (Jadidi et al., 2018). The pathogenesis of the reduced PWD after successful catheter ablation for AF is unclear. One possible hypothesis is that the reduced PWD may be caused by the electrical elimination and conduction of PV sleeves. Previous studies have demonstrated that myocardial sleeves in PVs may play an important role for the formation of the P wave (Ogawa et al., 2007; Okumura et al., 2007). Ogawa et al. (2007) reported the elimination of muscle sleeves inside the PVs resulted in shortening of the PWD and changes in the terminal portion of P‐wave morphology by analyzing a three‐dimensional computer simulation using an atrial cell model. Another study showed a decrease in biatrial conduction time after excluding the left PV activations, which was significantly correlated with reduced PWD following ablation (Okumura et al., 2007). Our study findings are in agreement with the abovementioned studies. Considering the former studies based on assessments performed by simulation or virtual methods, the present study provides stronger evidence for the evaluation of the association between actual PV reconnection and PWD in terms of clinical findings.

Interestingly, the PWD in the SAECG showed a significant decrease after ablation in patients with non‐PV reconnection, and conversely, the PWD in the SAECG increased in the PV reconnection group. The SAECG is a useful examination for PWD and provides a more accurate evaluation of atrial conduction; moreover, it has been assessed as a predictor of recurrence after catheter ablation for AF in several studies (Blanche et al., 2014, 2013; Kanzaki et al., 2016; Ogawa et al., 2007; Okumura et al., 2007; Van Beeumen et al., 2010). Likely, an increased PWD in SAECG might reflect a residual PV potential with delayed conduction between the LA and PV after the initial ablation session. In the presence of PV reconnection with a prolonged conduction, the PWD on SAECG could not be reduced after the initial ablation. Indeed, the intracardiac electrogram showed a significantly delayed PV potential from the beginning of the P wave in the circular mapping catheter, suggesting it may be responsible for the prolonged atrial activation as it increased the PWD observed on the SAECG after the initial session. This result was also supported by additional findings in the present study of some cases with PV reconnection presenting with a new emergent or delayed notched P wave on the ECG after the ablation.

In contrast, in the non‐PV reconnection group, the baseline notched P wave disappeared after the initial ablation in over half of the study population. The eliminated notched P wave following ablation might be due in part to the activation in the myocardial sleeves of the PV and PV junction including LA. Thus, the PWD and P‐wave morphology could be affected by the PV isolation. Nevertheless, not all individuals of the study population presented a diminished notched P wave after the ablation and only 21% of the patients exhibited this phenomenon in the validation cohort group in the present study. In this context, a previous study reported that approximately one‐fourth of the PVs were reconnected regardless of clinical recurrence after cryoballoon ablation for paroxysmal AF (Miyazaki et al., 2016). Some PV reconnections may have occurred that were not associated with any significant arrhythmogenic substrates in the recurrence‐free group after the ablation (Nakagawa et al., 2004). Considering the above silent PV reconnections without clinical recurrence, the prevalence of achieving a maximum PWD ≥ 5.0 ms and PWD in SAECG ≥ 3.5 ms as a predictor of non‐PV reconnection, 72% and 66%, might be estimated as adequate values in the validation cohort group. Moreover, besides the decrease in the PWD, the absence of notched P wave after the ablation could be also helpful to diagnose successful PV isolation.

However, Date et al. (2007) demonstrated that the excitation of PVs played a major role in the formation of the middle part of the P waves, and PV isolation did not change the PWD. Another study reported the terminal portion of P wave was comprised of several different regions of LA besides the PVs in an endocardial and epicardial mapping (Lemery et al., 2007). Indeed, some cases in our study showed a diminished spike wave at the middle portion of the P wave on the ECG after the ablation (cases 6 and 8) and not at the terminal end of the P wave. Some cases may have a broad substrate with conduction delay inside of PVs and LA antrum following a greater recovery of PWD after the PV isolation. Moreover, atrial scar and abnormal voltage in the atrium could affect conduction within the atrium, which may be associated with increased PWD and morphological changes, although the voltage map with the atrium and PVs was not assessed at preablation and postablation in this study. It should also be considered that the PWD might be affected by a recovery of remodeling in LA following ablation, although the LA diameter on the echocardiography between the baseline and repeat sessions did not change significantly. The detailed mechanism of the changes in PWD after the catheter ablation for AF would be verified by further well‐organized studies with multiple variables.

### 4.1 Study limitations

This study was retrospectively performed at a single institution. The sample size was relatively small due to the study design and strict exclusion criteria. Several baseline characteristics were different among the three groups. Since the P‐wave parameters and P‐wave morphology (notched/ not notched) were measured manually, the issue of reliability and reproducibility remains despite the confirmation of parameter agreement between inter‐ and intrainvestigators in this study. However, we also found similar results regarding the changes in PWD by calculating the SAECG, which can be obtained without manual measurement bias. Several R‐wave amplitudes and transitions on the ECG presented in Figures were different between the baseline and repeat procedure. The difference in lead placement could affect the P‐wave morphology and appearance and/or disappearance of P wave notching on the ECG recording. The calculated cutoff points of the PWD were based on patients who underwent a repeat procedure for recurrence. Therefore, we cannot ensure that these values could be applied similarly to patients without recurrence after the ablation. Moreover, absolute differences in PWD and signal‐averaged PWD after the ablation were found to be approximately 5–10 ms, which is a very small difference to distinguish. The cutoff values of PWD and signal‐averaged PWD based on the ROC curves were not very high and with a large potential error, requiring future development of a precise examination or more detailed analysis.

The appearance or disappearance of a notched P wave following ablation was not observed in all cases according to the PV reconnection. Indeed, in the non‐PV reconnection group without any evidence of a notched P wave at baseline, it was not possible to observe a diminished notched P wave after the ablation. Further, finding a notch in the P wave needs careful observation of the surface ECG with scaling, which may limit the universal clinical utility to some extent. For the validation cohort group, the lack of routine, standardized postablation rhythm assessment (i.e., frequent Holter, event monitoring at set intervals) could cause underestimation of the recurrence after the ablation.

## 5 CONCLUSIONS

The PWD was significantly changed after successful PV isolation for paroxysmal AF. The reverse dynamics of PWD after initial shortening directly following ablation were significantly associated with PV reconnection. The appearance or reduction of a notched P wave on the ECG was also characterized by PV reconnection. The elimination of PV sleeves during ablation may be responsible for the changes observed in P‐wave morphology on the ECG, which ultimately may be helpful to identify a successful PV isolation for paroxysmal AF. Our study results could represent a preparatory data for the future development of a noninvasive and simple examination to confirm PV isolation following ablation.

## CONFLICTS OF INTEREST

Drs. Yanagisawa and Shibata are affiliated with a department sponsored by Medtronic Japan. Other authors have no conflict of interest.

## References

[anec12712-bib-0001] Blanche, C. , Tran, N. , Carballo, D. , Rigamonti, F. , Burri, H. , & Zimmermann, M. (2014). Usefulness of P‐wave signal averaging to predict atrial fibrillation recurrences after electrical cardioversion. Annals of Noninvasive Electrocardiology, 19(3), 266–272. 10.1111/anec.12131 24397857 PMC6932666

[anec12712-bib-0002] Blanche, C. , Tran, N. , Rigamonti, F. , Burri, H. , & Zimmermann, M. (2013). Value of P‐wave signal averaging to predict atrial fibrillation recurrences after pulmonary vein isolation. Europace, 15(2), 198–204. 10.1093/europace/eus251 22941968

[anec12712-bib-0003] Caldwell, J. , Koppikar, S. , Barake, W. , Redfearn, D. , Michael, K. , Simpson, C. , … Baranchuk, A. (2014). Prolonged P‐wave duration is associated with atrial fibrillation recurrence after successful pulmonary vein isolation for paroxysmal atrial fibrillation. Journal of Interventional Cardiac Electrophysiology, 39(2), 131–138. 10.1007/s10840-013-9851-1 24306110

[anec12712-bib-0004] Calkins, H. , Hindricks, G. , Cappato, R. , Kim, Y.‐H. , Saad, E. B. , Aguinaga, L. , … Yamane, T. (2017). 2017 HRS/EHRA/ECAS/APHRS/SOLAECE expert consensus statement on catheter and surgical ablation of atrial fibrillation. Heart Rhythm: the Official Journal of the Heart Rhythm Society, 14(10), e275–e444. 10.1016/j.hrthm.2017.05.012 PMC6019327 28506916

[anec12712-bib-0005] Date, T. , Yamane, T. , Inada, K. , Matsuo, S. , Kanzaki, Y. , Miyanaga, S. , … Mochizuki, S. (2007). The effects of pulmonary vein isolation on the morphology of p waves: The contribution of pulmonary vein muscle excitation to the formation of p waves. Pacing and Clinical Electrophysiology, 30(1), 93–101. 10.1111/j.1540-8159.2007.00570.x 17241321

[anec12712-bib-0006] Haïssaguerre, M. , Jaïs, P. , Shah, D. C. , Takahashi, A. , Hocini, M. , Quiniou, G. , … Clémenty, J. (1998). Spontaneous initiation of atrial fibrillation by ectopic beats originating in the pulmonary veins. New England Journal of Medicine, 339(10), 659–666. 10.1056/NEJM199809033391003 9725923

[anec12712-bib-0007] Jadidi, A. , Muller‐Edenborn, B. , Chen, J. , Keyl, C. , Weber, R. , Allgeier, J. , … Arentz, T. (2018). The duration of the amplified sinus‐P‐Wave identifies presence of left atrial low voltage substrate and predicts outcome after pulmonary vein isolation in patients with persistent atrial fibrillation. JACC: Clinical Electrophysiology, 4(4), 531–543. 10.1016/j.jacep.2017.12.001 30067494

[anec12712-bib-0008] Kanzaki, Y. , Inden, Y. , Ando, M. , Kamikubo, Y. , Ito, T. , Mizutani, Y. , … Murohara, T. (2016). An ECG index of P‐Wave force predicts the recurrence of atrial fibrillation after pulmonary vein isolation. Pacing and Clinical Electrophysiology, 39(11), 1191–1197. 10.1111/pace.12956 27723112

[anec12712-bib-0009] Kim, T. H. , Park, J. , Uhm, J. S. , Joung, B. , Lee, M. H. , & Pak, H. N. (2017). Pulmonary vein reconnection predicts good clinical outcome after second catheter ablation for atrial fibrillation. Europace, 19(6), 961–967. 10.1093/europace/euw128 27256420

[anec12712-bib-0010] Lemery, R. , Birnie, D. , Tang, A. S. , Green, M. , Gollob, M. , Hendry, M. , & Lau, E. (2007). Normal atrial activation and voltage during sinus rhythm in the human heart: An endocardial and epicardial mapping study in patients with a history of atrial fibrillation. Journal of Cardiovascular Electrophysiology, 18(4), 402–408. 10.1111/j.1540-8167.2007.00762.x 17394455

[anec12712-bib-0011] Miyazaki, S. , Taniguchi, H. , Hachiya, H. , Nakamura, H. , Takagi, T. , Hirao, K. , & Iesaka, Y. (2016). Clinical recurrence and electrical pulmonary vein reconnections after second‐generation cryoballoon ablation. Heart Rhythm: the Official Journal of the Heart Rhythm Society, 13(9), 1852–1857. 10.1016/j.hrthm.2016.05.025 27241352

[anec12712-bib-0012] Nakagawa, H. , Aoyama, H. , Beckman, K. J. , Po, S. S. , Wu, R. , Lockwood, D. , … Jackman, W. M. (2004). Relation between pulmonary vein firing and extent of left atrial‐pulmonary vein connection in patients with atrial fibrillation. Circulation, 109(12), 1523–1529. 10.1161/01.CIR.0000121745.13435.E0 15023867

[anec12712-bib-0013] Nakatani, Y. , Sakamoto, T. , Mizumaki, K. , Nishida, K. , Kataoka, N. , Tsujino, Y. , … Inoue, H. (2016). Coefficient of variation of P‐wave duration is a novel atrial heterogeneity index to predict recurrence of atrial fibrillation after catheter ablation. Journal of Cardiovascular Electrophysiology, 27(5), 542–548. 10.1111/jce.12920 26756553

[anec12712-bib-0014] Ogawa, M. , Kumagai, K. , Vakulenko, M. , Yasuda, T. , Siegerman, C. , Garfinkel, A. , … Saku, K. (2007). Reduction of P‐wave duration and successful pulmonary vein isolation in patients with atrial fibrillation. Journal of Cardiovascular Electrophysiology, 18(9), 931–938. 10.1111/j.1540-8167.2007.00890.x 17655679

[anec12712-bib-0015] Okumura, Y. , Watanabe, I. , Ohkubo, K. , Ashino, S. , Kofune, M. , Hashimoto, K. , … Saito, S. (2007). Prediction of the efficacy of pulmonary vein isolation for the treatment of atrial fibrillation by the signal‐averaged P‐wave duration. Pacing and Clinical Electrophysiology, 30(3), 304–313. 10.1111/j.1540-8159.2007.00670.x 17367349

[anec12712-bib-0016] Platonov, P. G. (2012). P‐wave morphology: Underlying mechanisms and clinical implications. Annals of Noninvasive Electrocardiology, 17(3), 161–169. 10.1111/j.1542-474X.2012.00534.x 22816534 PMC6932587

[anec12712-bib-0017] Shah, S. , Barakat, A. F. , Saliba, W. I. , Abdur Rehman, K. , Tarakji, K. G. , Rickard, J. , … Hussein, A. A. (2018). Recurrent atrial fibrillation after initial long‐term ablation success: electrophysiological findings and outcomes of repeat ablation procedures. Circulation: Arrhythmia and Electrophysiology, 11(4), e005785 10.1161/CIRCEP.117.005785 29654129

[anec12712-bib-0018] Sohara, H. , Ohe, T. , Okumura, K. , Naito, S. , Hirao, K. , Shoda, M. , … Aonuma, K. (2016). HotBalloon ablation of the pulmonary veins for paroxysmal AF: A multicenter randomized trial in Japan. Journal of the American College of Cardiology, 68(25), 2747–2757. 10.1016/j.jacc.2016.10.037 28007137

[anec12712-bib-0019] Van Beeumen, K. , Houben, R. , Tavernier, R. , Ketels, S. , & Duytschaever, M. (2010). Changes in P‐wave area and P‐wave duration after circumferential pulmonary vein isolation. Europace, 12(6), 798–804. 10.1093/europace/eup410 20047928

[anec12712-bib-0020] Yanagisawa, S. , Inden, Y. , Fujii, A. , Kamikubo, Y. , Kanzaki, Y. , Ando, M. , … Murohara, T. (2018). Assessment of autonomic nervous system modulation after novel catheter ablation techniques for atrial fibrillation using multiple short‐term electrocardiogram recordings. Journal of Interventional Cardiac Electrophysiology, 51(1), 35–44. 10.1007/s10840-017-0295-x 29110167

[anec12712-bib-0021] Zucchelli, G. , Sirico, G. , Rebellato, L. , Marini, M. , Stabile, G. , Del Greco, M. , … Bongiorni, M. G. (2018). Contiguity between ablation lesions and strict catheter stability settings assessed by VISITAG(TM) module improve clinical outcomes of paroxysmal atrial fibrillation ablation‐ results from the VISITALY study. Circulation Journal, 82(4), 974–982. 10.1253/circj.CJ-17-0421 29415917

