# Supplementary material for: Electrocardiogram characteristics of P wave associated with successful pulmonary vein isolation in patients with paroxysmal atrial fibrillation: Significance of changes in P‐wave duration and notched P wave
Source: Ann Noninvasive Electrocardiol. 2019 Sep 30;25(2):e12712. doi: 10.1111/anec.12712 (PMC7358886; doi:10.1111/anec.12712)
Supplement: Supplementary file 1 [file ANEC-25-e12712-s001.pdf]

Supplemental Figure 1

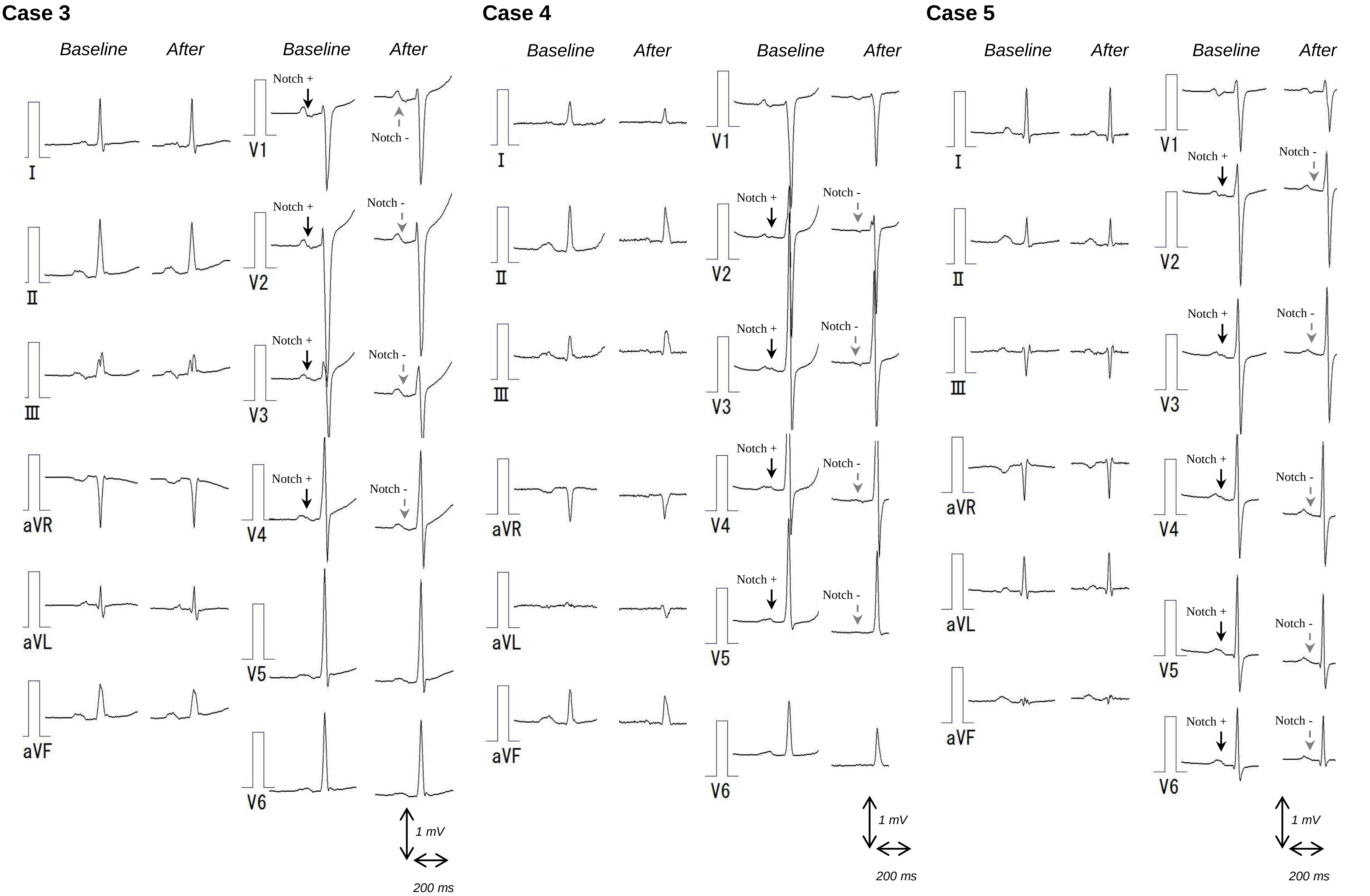

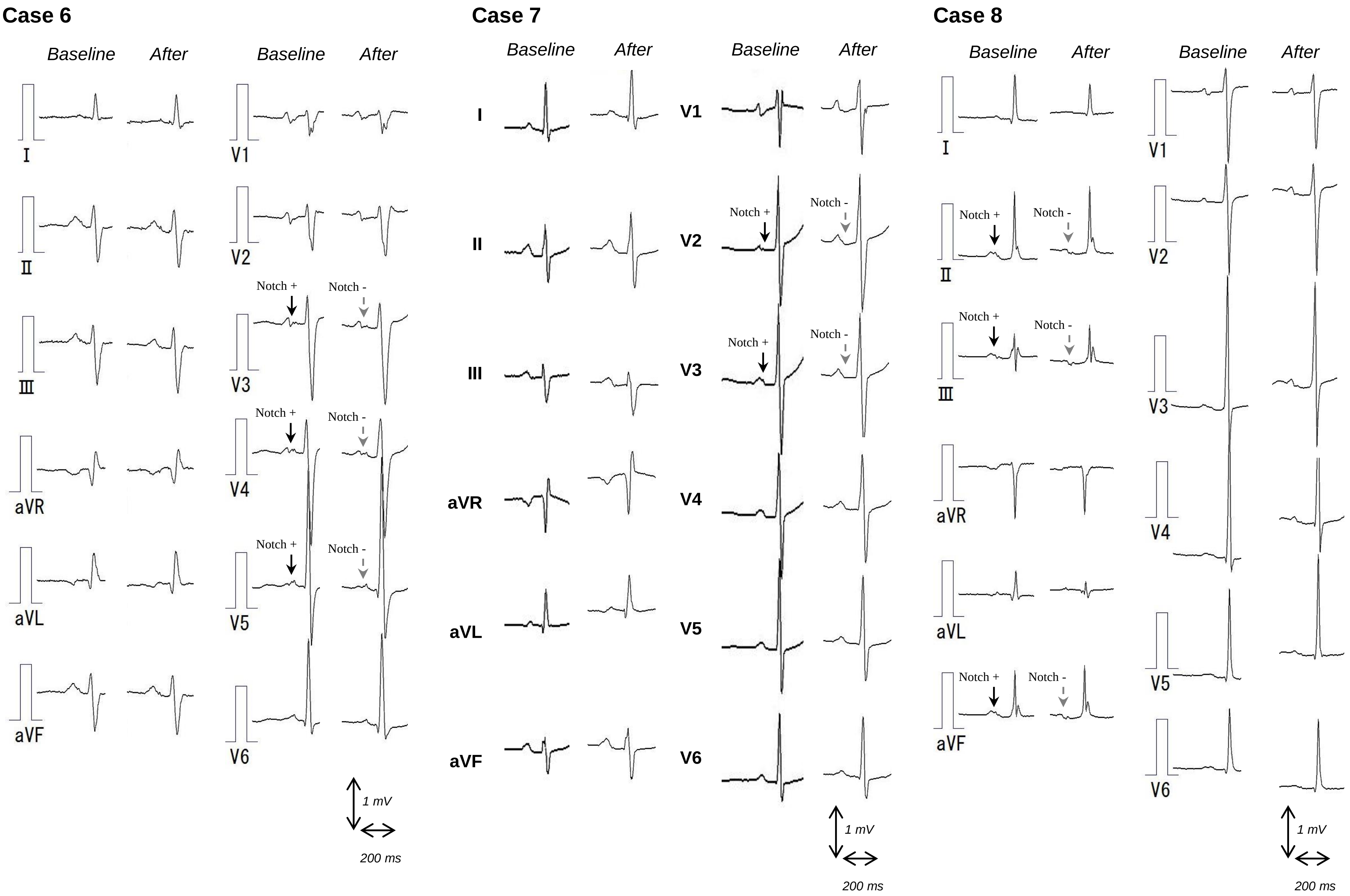

Case 6
Baseline
After
Baseline
After
I
II
III
aVR
aVL
aVF
V1
V2
V3
V4
V5
V6
Notch +
Notch -
Notch +
Notch -
Notch +
Notch -
1 mV
200 ms
Case 7
Baseline
After
Baseline
After
I
II
III
aVR
aVL
aVF
V1
V2
V3
V4
V5
V6
Notch +
Notch -
Notch +
Notch -
1 mV
200 ms
Case 8
Baseline
After
Baseline
After
I
II
III
aVR
aVL
aVF
V1
V2
V3
V4
V5
V6
Notch +
Notch -
Notch +
Notch -
Notch +
Notch -
1 mV
200 ms

Supplemental Figure 1

Changes in P-wave morphology on the electrocardiogram following ablation in patients without PV reconnection at the time of the repeat procedure (case 3-8).

In case 3 to 5, and case 7, the baseline electrocardiogram showed a notched P-wave in the precordial leads. At the repeat ablation procedure, all the notched P-waves disappeared. In case 6, there were two spike waves after the deep notched P-wave at the baseline in the V2-4 leads. After the ablation, the first spike wave disappeared after the deep notched P-wave, but the second spike wave remained. The notch between the first and second spike waves was abolished at the time of repeat procedure. Case 8 represented three spike waves with 2 notches in the P-wave in the inferior leads at baseline. Following ablation, the second spike wave disappeared with the elimination of the first notched P-wave. However, the first and third spike waves remained.

Supplemental Figure 2

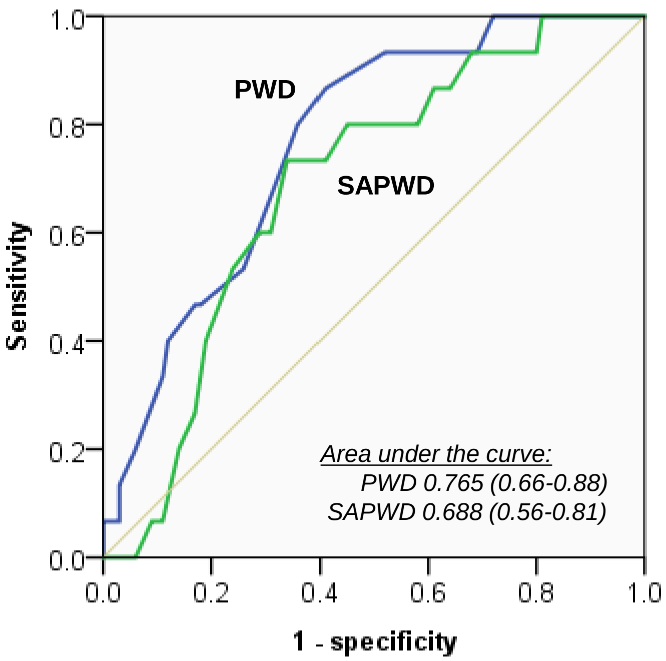

A

**PWD**	Sensitivity	1-specificity
-11.0000	1.000	0.890
-9.0000	1.000	0.870
-7.0000	1.000	0.820
-5.0000	1.000	0.770
-3.0000	1.000	0.720
-1.0000	0.933	0.690
1.0000	0.933	0.560
3.0000	0.933	0.520
**5.0000**	**0.867**	**0.410**
7.0000	0.800	0.360
9.0000	0.533	0.260
10.5000	0.467	0.180
11.5000	0.467	0.170
13.0000	0.400	0.120
15.0000	0.333	0.110

B

**SAPWD**	Sensitivity	1-specificity
-3.5000	0.800	0.540
-2.5000	0.800	0.490
-1.5000	0.800	0.470
-.5000	0.800	0.450
.5000	0.733	0.410
1.5000	0.733	0.400
2.5000	0.733	0.380
**3.5000**	**0.733**	**0.340**
4.5000	0.600	0.310
5.5000	0.600	0.290
7.0000	0.533	0.240
8.5000	0.400	0.190
9.5000	0.333	0.180
10.5000	0.267	0.170
12.5000	0.200	0.140

Supplemental Figure 2

The receiver operating characteristic curve analyses of maximum PWD and SAPWD for the prediction of non-PV reconnection after the ablation

PV, pulmonary vein; PWD, P-wave duration; SAPWD, signal-averaged P-wave duration.
